# Harmonic information transitions of spatiotemporal metasurfaces

**DOI:** 10.1038/s41377-020-00441-1

**Published:** 2020-12-14

**Authors:** Haotian Wu, Xin Xin Gao, Lei Zhang, Guo Dong Bai, Qiang Cheng, Lianlin Li, Tie Jun Cui

**Affiliations:** 1 State Key Laboratory of Millimetre Waves, Southeast University, 210096 Nanjing, China; 2 Institute of Electromagnetic Space, Southeast University, 210096 Nanjing, China; 3 State Key Laboratory of Advanced Optical Communication Systems and Networks, Department of Electronics, Peking University, 100871 Beijing, China

**Keywords:** Metamaterials, High-harmonic generation

## Abstract

Facilitated by ultrafast dynamic modulations, spatiotemporal metasurfaces have been identified as a pivotal platform for manipulating electromagnetic waves and creating exotic physical phenomena, such as dispersion cancellation, Lorentz reciprocity breakage, and Doppler illusions. Motivated by emerging information-oriented technologies, we hereby probe the information transition mechanisms induced by spatiotemporal variations and present a general model to characterize the information processing capabilities of the spatiotemporal metasurface. Group theory and abstract number theory are adopted through this investigation, by which the group extension and independent controls of multiple harmonics are proposed and demonstrated as two major tools for information transitions from the spatiotemporal domain to the spectra-wavevector domain. By incorporating Shannon’s entropy theory into the proposed model, we further discover the corresponding information transition efficiencies and the upper bound of the channel capacity of the spatiotemporal metasurface. The results of harmonic information transitions show great potential in achieving high-capacity versatile information processing systems with spatiotemporal metasurfaces.

## Metamaterials: Heightened harmonics from moving metasurfaces

The generation of light harmonics by rapidly-changing metasurfaces could provide exciting new methods of information processing. Metasurfaces made from metal or semiconducting nanostructures have opened up new ways of manipulating electromagnetic waves, but most are static in nature. More recently, spatiotemporal metasurfaces have emerged, in which the ‘meta-atoms’ that affect the waves can be rearranged periodically, through electrical or mechanical forcing. Now, Tie Jun Cui and co-workers at Southeast University in Nanjing and Peking University in Beijing have explained how several useful information transitions can be induced by spatiotemporal metasurfaces. Through a theoretical model and a proof-of-principle demonstration with microwaves, the researchers show how spatiotemporal metasurfaces can generate multiple harmonics from incoming waves. These harmonics represent processed information that could benefit new technologies such as intelligent computational imaging and sixth-generation (6G) wireless networks.

## Introduction

Photonic state transitions, akin to the Stokes Raman effect with inelastic scattering, require phase matching between the initial and final states of electromagnetic waves. In bulk optics, this can be achieved by perturbing the dielectric constant of the medium with mechanical vibrations or optical excitations^1–3^. Mirroring the hierarchy of photonic transitions in perturbed media, spatiotemporal metasurfaces, with reduced dimensionality and enhanced versatility, have prompted a similar route towards manipulating the state of electromagnetic waves via on-demand spatial and temporal variations. In contrast to the case of the stable metasurface, the meta-atoms of the spatiotemporal metasurface are hybridized with electrically or optically controllable elements, such that the metasurface can be considered an optical platform capable of spatiotemporally rearrangement^4–9^. Electromagnetic waves propagating through such a platform manifest exotic physical phenomena and intriguing functionalities, such as engineering angular dispersion, breaking Lorentz reciprocity, and shifting the Doppler frequency, which promise to create economical in situ optical systems with conformal integration^10–17^.

In line with the study of spatiotemporal metasurfaces, efforts have been made to integrate metasurfaces with digital and information science, enabling the emergence of digital coding metasurfaces, information metasurfaces, and intelligent metasurfaces^18–25^. Recently, a number of information-based metasurfaces have been developed, enabling the flexible harvesting of photonic information with self-adaptive radiation formation, neural-network-based computational imagers, and deep-learning-induced microwave cameras^26–28^. Despite these advances, there has been no scheme that provides a fundamental model to study and characterize the information transitions induced by the spatiotemporal metasurface.

In this article, we adopt the concepts of group theory and abstract number theory to analyze the information transitions of the spatiotemporal metasurface. A proof-in-principle experiment is performed in the microwave regime for verification. Additionally, by incorporating Shannon’s information entropy theory, we further discover the upper bound of the channel capacity of the spatiotemporal metasurface and demonstrate the connection between the channel capacity and the intensity of the converted field.

## Results

### Characterization of spatiotemporal metasurface

**Fig. 1:**
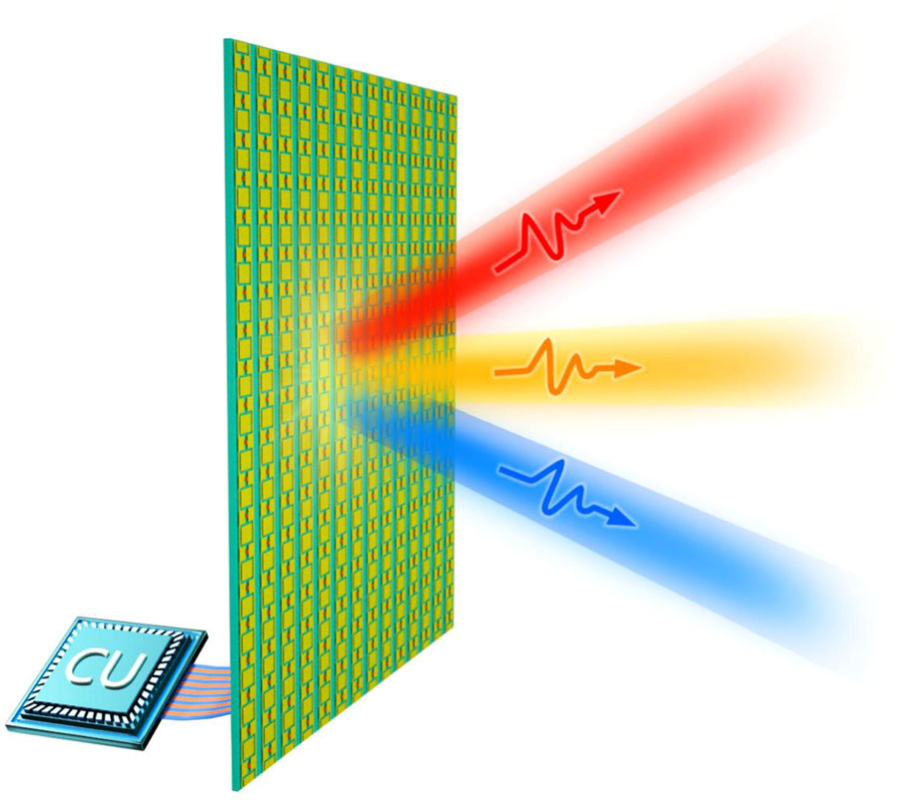
The conceptual illustration of harmonic information transitions induced by the spatiotemporal metasurface. The responses of the meta-atoms can be rapidly switched by an FPGA with engineered spatiotemporal variations, generating four-wavevector transitions of electromagnetic waves in the far-field region

Driven by customized and high-speed space-time variations of the metasurface, the photonic states of the impinging electromagnetic waves can be tailored with many degrees of freedom. Specifically, the interactions between the normally incident monochromatic electromagnetic waves and periodic-modulated spatiotemporal metasurface generate photonic four-wavevector ($\omega ,\,k_{{x}},\,k_{{y}},\,k_{{z}}$) transitions in the far-field region (as is sketched in Fig. 1), and the transition magnitudes can be expressed as^5,7^:

1 $$f(\omega ,{\mathbf{k}}) = \mathop {\sum}\limits_{r = 1}^P {\mathop {\sum}\limits_{s = 1}^Q {G_{rs}(\omega ) \cdot I_{rs}^{\omega _0}(k_x,k_y) \cdot \exp [j(rk_x{\mathrm{d}}x + sk_y{\mathrm{d}}y)]} }$$

where $I_{rs}^{\omega _0}$ is the far-field pattern pertaining to the *rs*th meta-atom computed at the central frequency *f*_0_, and $G_{rs}(\omega )$ represents the frequency-domain response of the *rs*th meta-atom, for which the dynamic modulation scheme introduces broad spectral components. Notably, the frequency-domain response term $G_{rs}(\omega )$ can be reformulated as an infinite train of pulses at discrete harmonic frequencies ($\omega = \omega _0 + {{m}}\omega _1$) as:

2 $$G_{rs}^m = G_{rs}\left( {\omega _0 + m\omega _1} \right) = \frac{1}{L}\mathop {\sum}\limits_{i = 0}^{L - 1} {C_{rs}^i} {\mathrm{sinc}}\left( {\frac{{m\pi }}{L}} \right){\mathrm{exp}}\left[ {\frac{{ - jm\left( {2i + 1} \right)\pi }}{L}} \right]$$

where $\omega _1 = 2\pi /L\tau$ is the basic modulation frequency, *τ* is the unit time duration of the input pulse, and *m* is an arbitrary integer that represents the discrete harmonic frequency (harmonic index). The term $C_{rs}^i$ represents the complex amplitude response of the *rs*th meta-atom at the *i*th time interval in one period, and the term *L* represents the periodicity length of the input modulation sequence.

Note that the reference phase state of zero of the meta-atom can be set freely at different harmonics. Therefore, to simplify the expression of the spectral responses of the meta-atom, it is helpful to adopt frequency-dependent gauge transformations to the output responses of the meta-atom at the *m*th harmonic frequency as $H_{rs}^m = H_{rs}\left( {\omega _0 + m\omega _1} \right) = G_{rs}\left( {\omega _0 + m\omega _1} \right){\mathrm{exp}}\left( {j\frac{{m\pi }}{L}} \right)$, in which the spectra-isolated global gauge choice $\left( {\exp \left( {j\frac{{m\pi }}{L}} \right)} \right)$ has no effect on the interactions between the electromagnetic waves and the spatiotemporal metasurface. Consequently, the effective electric field response at the *m*th harmonic frequency can be expressed with a more symmetrical form as:

3 $$H_{rs}^m = \frac{1}{L}\mathop {\sum}\limits_{i = 0}^{L - 1} {C_{rs}^i} {\mathrm{sin}}c\left( {\frac{{m\pi }}{L}} \right)\exp \left( { - j\frac{{2im\pi }}{L}} \right)$$

Notably, Eq. (3) implies that the infinite spectral responses ($H_{rs}^m$) share the translational linear dependency:

4 $$H_{rs}^{m + cL} = \frac{1}{L}\mathop {\sum}\limits_{i = 0}^{L - 1} {C_{rs}^i} \cdot {\mathrm{sin}}c\left[ {\frac{{(m + cL)\pi }}{L}} \right]\exp \left[ { - j\frac{{2(m + cL)i\pi }}{L}} \right] = ( - 1)^c \cdot \frac{m}{{m + Lc}}H_{rs}^m$$

where $c \in {{Z}}$. Therefore, it is helpful to group the linearly dependent spectral components together and partition the harmonic components into *L* distinctive sets. Accordingly, each linearly dependent set contains infinite spectral components and can be denoted as $\{ H_{rs}^{m|L}\}$, where $m|L$ is the abbreviation for *m* modulo *L*. We remark that the set $\{ H^0\}$ contains a unique non-zero component of $H^0$, and all other spectral responses in the set $\{ H^0\}$ are equal to zero. Therefore, the spectral responses of the spatiotemporal metasurface from the 0th to the *L* − 1th harmonic frequencies can be analyzed, from which the spectral response information at other harmonics can be obtained from Eq. (4).

To simplify the notation, the input temporal sequence of the meta-atom in one temporal period is denoted as $C_{rs} = \left( {C_{rs}^0,C_{rs}^1 \ldots C_{rs}^{L - 1}} \right)$, and the corresponding spectral responses of the meta-atom at the 0th to the *L* − 1th harmonics are denoted as $H_{rs} = \left( {H_{rs}^0,H_{rs}^1 \ldots H_{rs}^{L - 1}} \right)$.

Notably, the output spectral responses of the meta-atom from the 0th to the *L* − 1th harmonics $H_{rs}$ are related to the input temporal sequence $C_{rs}$ by the non-degenerate matrix transform as $H_{rs} = {\mathrm{{\Lambda}}} \times {\mathrm{V}} \times C_{rs}$, where ${\mathrm{{\Lambda}}}_m^n = \frac{1}{L}\delta _m^n{\mathrm{sin}}c\left( {\frac{{m\pi }}{L}} \right)$ is a non-degenerate diagonal matrix of order *L* and V is the order-*L* unitary Vandermonde matrix that can be denoted as ${\mathrm{V}}_m^n = \zeta _L^{ - mn}$ ($n,\,m = 0,\,1, \ldots ,L - 1$). The term $\zeta _L$ represents the *L*th complex root of unity, which can be expressed as $\zeta _L = {\mathrm{exp}}(i2\pi /L)$. The above non-degenerate matrix product imposes a bijection relation ($C_{rs} \,\mapsto\, H_{rs}$) to the input temporal sequence and the output spectral responses.

From the information perspective, the spatiotemporal metasurface can be considered the information processer to transform the input spatiotemporal modulated digital information (spatiotemporal sequence of the metasurface) to the spatial-spectral response information at harmonic frequencies. Subsequently, the spatial-spectral response distribution of the metasurface can be used to manipulate the transmission of the electromagnetic waves in the far-field region, thus yielding the information transitions from the spatial-spectral domain to the spectral-wavevector domain.

Ideally, due to the bijection relation between the input spatiotemporal sequence and spatial-spectral responses $\left( {C_{rs} \,\mapsto\, H_{rs}} \right)$ of the metasurface, it is possible to transform all of the input spatiotemporal information to the spectral-wavevector information. However, we note that not all realizable spatiotemporal sequences are preferred to control the flow of electromagnetic waves. Specifically, phase modulation schemes are mostly used in manipulating the transmission of electromagnetic waves. Therefore, in this work, it is required that the spectral responses of each meta-atom at the required harmonic frequency/frequencies are amplitude-invariant (phase-modulated). Consequently, the corresponding information transition efficiency of the spatiotemporal metasurface might be compromised.

In the following, we analyze the main characteristics of the spatiotemporal metasurface and demonstrate a natural way to generate amplitude-invariant uniform phase-modulated spectral responses.

Notably, two major aspects of the spatiotemporal metasurfaces that are not attainable by the spatially modulated metasurface alone (i.e., group extensions and the independent controls of multiple harmonics) are demonstrated. Specifically, the group extension mechanism can extend the output phase states of each meta-atom by a factor of *q*, which can provide more accurate manipulation of the electromagnetic information without complicated integrations. The independent controls of multiple harmonics can open up new possibilities for metasurface-based multitasking, by which electromagnetic information can be parallelly processed with frequency gapped channels.

### Group extension mechanisms

Field extension^29^ has a fundamental role in abstract algebra. For instance, under the usual notions of addition and multiplication, the complex numbers are the extension fields of the real numbers. In turn, the real numbers are a subfield of the complex numbers. In analogy to the field extension, we show that the possible phase responses of each meta-atom can be considered as a symmetry group and that the number of output spectral responses at the *m*th harmonic frequency can be extended due to the dynamical variations, in which the state extension mechanisms can be characterized by the group extensions^29^. For the first time, we show that the input uniform *N* phase states of each meta-atom are extended to $N \times q$ phase states at the *m*th harmonic frequency when specific modulation schemes are adopted. The state extension factor of the spatiotemporal metasurface (*q*) is a function of the harmonic index (*m*), the number of input phase states (*N*), and the length of temporal periodicity (*L*). In the remaining discussions, the lower index of meta-atom (*rs*) is eliminated when no confusion is possible.

Due to the advantages of the phase modulations in manipulating the transmission of the electromagnetic waves, the temporal responses of the meta-atoms are required to be phase-modulated. Accordingly, once the number of modulated phase states (*N*) of the spatiotemporal metasurface is determined, the possible electric field response of the meta-atom at the *i*th temporal interval ($C^i$) can be expressed with the complex unit $C^i = \zeta _N^u$ (*u* = 0, 1 … *N* − 1). To simplify the descriptions, the notation $C^i(u)$ is introduced, which implies that the response of the meta-atom at the *i*th temporal interval is $C^i = \zeta _N^u$.

**Fig. 2:**
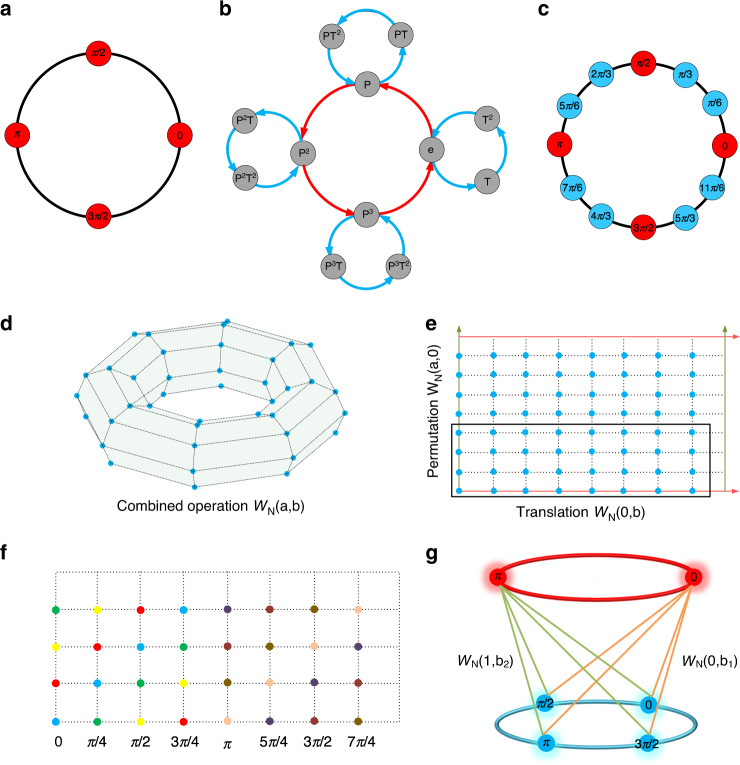
Principles of harmonic information transitions with a spatiotemporal metasurface. **a** The possible input phase states of the meta-atom with $N = 4$. **b** Cayley diagram of the product group ${\mathrm{Z}}_N \times {\mathrm{Z}}_q$ that generates $N \times q$ output phase states. **c** The generated spectral states of the meta-atom, in which the corresponding symmetry is described by the group ${\mathrm{Z}}_{N \times q}$. **d** The group structure of the combined operations $W_N\left( {a,b} \right)$. **e** Unfolded representation of the combined operations in the two-dimensional space, in which the colored arrows denote the equivalent relations of the operations. The generated distinctive temporal sequences are contained in the black-line box. **f** Spectral response states of the generated sequences, in which differently colored circles represent different phase responses. **g** Schematic of independent control of spectral responses of the meta-atom at two harmonics, in which the line connects the spectral responses generated by the same temporal modulations

It should be noted that the possible input phase states are rotationally symmetrically distributed on the complex plane, by which the corresponding symmetry can be described by the additive group ${\mathrm{Z}}_N$. For instance, the symmetry of input phase states with $N = 4$ can be described by the group ${\mathrm{Z}}_4$ (shown in Fig. 2a), for which any one of the four phase states can be gauged out to be unity (phase response state of zero).

To further explore the property of spectral responses of the meta-atom, tensor notation is adopted, by which the output spectral responses of each meta-atom can be expressed with the *L*-dimensional tensor products as:

5 $$H^m = {\Lambda}_i^mV_j^iC^j$$

where the Einstein notation is adopted such that the repeated indices indicate summations (*m* = 0, 1, …, *L* − 1). Notably, the above Einstein notation implies the summation of indexed terms from 0 to *L* − 1 rather than 1 to *L*.

Here, we first analyze the scenario when the spectral response of the meta-atom at the *m*th harmonic frequency is non-zero ($H^m\, \ne\, 0$). Due to the ${\mathrm{Z}}_N$ symmetry of the input phase states, it can be verified that the permutation of all elements in the phase states would generate a phase shift to the spectral responses of the (*rs*th) meta-atom. More specifically, a collection of permutations of the input temporal sequence $\left( {C = C^0\left( u \right),C^1\left( v \right) \ldots C^{L - 1}\left( w \right)} \right)$ from $C^0(u)$ to $C^0\left( {\left( {u + a} \right)|N} \right)$, $C^1(v)$ to $C^1((v + a)|N)$ …, and $C^{L - 1}(w)$ to $C^{L - 1}((w + a)|N)$ would generate a phase shift of ${\upzeta}_N^a$ to the spectra responses of the meta-atom at each harmonic frequency. Alternatively, the above rearrangements of the input phase states can be considered group actions, such that the generated phase shift effect can be expressed with a more compact form as $P_N\left( a \right)H^m = {\mathrm{{\Lambda}}}_i^mV_j^iP_N\left( a \right)C^j = \zeta _N^aH^m$, where $P_N\left( {\mathrm{a}} \right)$ $(P_N\left( {\mathrm{a}} \right) = P^a)$ denotes the above collection of permutation operations that acted on the temporal sequence.

Evidently, the possible output phase states of each meta-atom at the 0th to *L* − 1th harmonics must contain the original *N* phase states (0, $\zeta _N^1$, $\zeta _N^2$…$\zeta _N^{N - 1}$) as well, as long as the corresponding spectral response of the meta-atom is non-zero. In other words, the output spectral responses of the meta-atom must contain the same symmetry of ${\mathrm{Z}}_N$ as the input response states.

According to Eq. (4), the spectral responses are also affected by the weighted summation factor introduced by each row of the Vandermonde matrix V, such that the output spectral responses of the meta-atom might contain other symmetries as well. To simplify further analysis, the *m*th row of the Vandermonde matrix is denoted as ${\mathrm{{\Omega}}}\left( m \right)$, where ${\mathrm{{\Omega}}}\left( m \right) = \left( {\zeta _L^0,\,\zeta _L^{ - m}\, \ldots \,\zeta _L^{ - m(L - 1)}} \right)$. Subsequently, the second operation $T\left( b \right)$ $\left( {T\left( b \right) = T^b} \right)$, which translates the input temporal response sequence $C = \left( {C^0,C^1 \ldots C^{L - 1}} \right)$ with $b$ unit intervals as $T(b)\left( {C^0,C^1 \ldots C^{L - 1}} \right) = \left( {C^{ - b},C^{ - b + 1} \ldots C^{ - b + L - 1}} \right)$, is introduced and defined as the translation operator. As a result, the spectral responses of the meta-atom acted by the translation operator generate a frequency-dependent phase shift of $\zeta _L^{ - bm}$, which can be expressed in tensor form as:

6 $$T(b)H^m = {\Lambda}_j^mV_i^jT(b)C^i = \zeta _L^{ - bm}H^m$$

Now, two operations, permutation and translation, have been found, which account for the phase shift of the output spectral responses of the meta-atoms. Additionally, it can be verified that the above two operations commute to each other. Therefore, these two operations can be composited as a combined operator (operation) as $W_N(a,b) = P_N(a) \times T(b)$. The combined operator acting on the temporal sequence of the meta-atom indicates the translation of the temporal sequence with *b* units at first and then permutes all phase states of the sequence from $C^0(u)$ to $C^0((u + a)|N)$, $C^1(v)$ to $C^1((v + a)|N)$…$C^{L - 1}(w)$ to $C^{L - 1}((w + a)|N)$. As a result, the spectral responses of the meta-atom generate a frequency-dependent composited phase shift of $\zeta _N^a \times \zeta _L^{ - bm}$, and the number of generated phase states at the *m*th harmonic frequency can be denoted as $N \times q$, where *q* is denoted as the group extension factor and can be derived as $q = L/[{\mathrm{gcd}}(L,m) \cdot {\mathrm{gcd}}(N,L/{\mathrm{gcd}}(L,m))]$ (the term “gcd” is the abbreviation for the greatest common divisor). The detailed derivations can be found in the Supplementary Information.

The symmetry of the combined operations can be described by the product group of ${\mathrm{Z}}_N \times {\mathrm{Z}}_L$, which can be denoted as the fundamental group. That is, the topological structure of the combined operations corresponds to a torus made of $N \times L$ lattice points, as shown in Fig. 2d. The torus can be further unfolded in a 2-dimensional space to a parallelogram, as shown in Fig. 2e.

We remark that not all elements of the combined operations are required to generate the extended spectral states. Specifically, the operations that generate the $N \times q$ output phase states are composed of *N* possible permutation operations followed by *q* possible translation operations, in which the corresponding symmetry can be described by the product group ${\mathrm{Z}}_N \times {\mathrm{Z}}_q$, as shown in Fig. 2b. Notably, the group ${\mathrm{Z}}_N \times {\mathrm{Z}}_q$ that generates $N \times q$ distinctive output phase states is an extension of the group ${\mathrm{Z}}_N$ that generates $N$ input phase states. Therefore, the group extension mechanism of the spatiotemporal metasurface can be denoted as ${\mathrm{Z}}_N\mathop { \to }\limits^{{\mathrm{extended}}\,{\mathrm{to}}} {\mathrm{Z}}_N \times {\mathrm{Z}}_q$.

For instance, suppose $N = 4$ and $L = 6$, such that the group extension factor can be derived as $q = 3$ at the 1st harmonic. As discussed above, permutation operations alone correspond to the group ${\mathrm{Z}}_4$, in which the generator of the group is the permutation operation (blue arrow) and the group elements can be denoted as *e*, *P*, *P*^2^, and *P*^3^ (shown in Fig. 2b). Consequently, four distinctive phase states uniformly covering the $2{\uppi}$ radians are generated when adopted with these operations, as shown in Fig. 2a.

On the other hand, when both the permutation and translation operations are considered, $N \times q$ ($4 \times 3$) output phase states uniformly covering $2{\uppi}$ radians are generated, as shown in Fig. 2c. The operations generated above $N \times q$ ($4 \times 3$) phase states can be described by the direct product of *N* (4) permutation operations ($e,\,P,\,P^2,P^3$) and *q* (3) translation operations ($e,\,T,\,T^2$), in which the generators of the product group are the permutation (blue arrow) and translation (red arrow) operations, respectively (shown in Fig. 2b).

Notably, there exists a bijection relation between the group elements of ${\mathrm{Z}}_N \times {\mathrm{Z}}_q$ and the generated $N \times q$ output phase states. For instance, it can be verified that the operation “$P^2T$” (shown in Fig. 2b) corresponds to a phase state of $2{\uppi}/3$ (shown in Fig. 2c), which indicates that performing the translation operation once, followed by two permutation operations, generates a phase state of $2{\uppi}/3$ to the meta-atom at the 1st harmonic frequency.

**Fig. 3:**
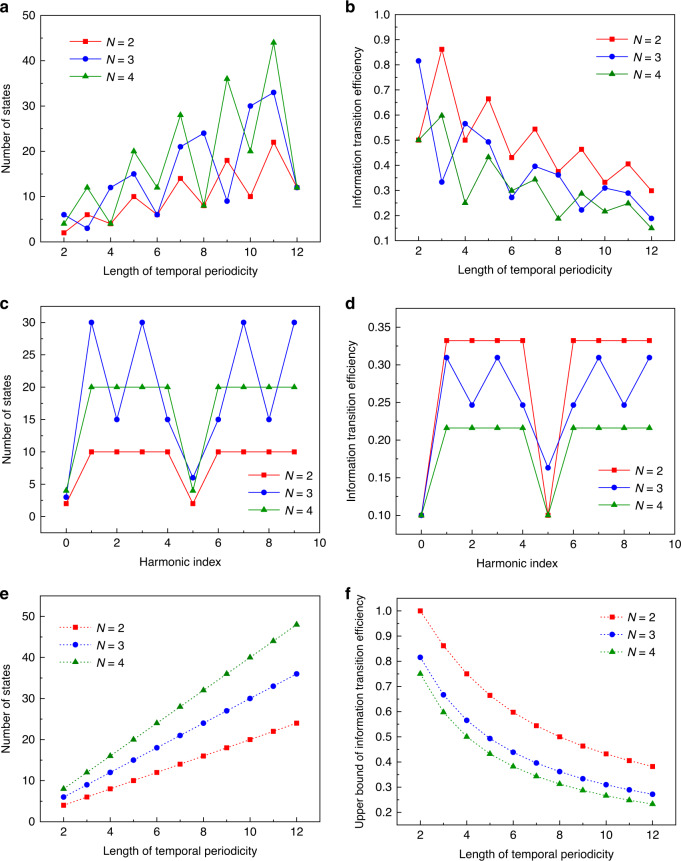
Generated spectral states of the meta-atom and the corresponding information transition efficiency. **a**, **b** Number of generated spectral states of a single meta-atom at the 1st harmonic frequency and the corresponding information transition efficiency with respect to different temporal periodicity and input states. **c**, **d** Number of generated states of a single meta-atom and the corresponding information transition efficiency with respect to different input states and harmonic index when $L = 10$. **e**, **f** Upper bound of the generated spectral states of a single meta-atom and the corresponding information transition efficiency

Although the output phase states of each meta-atom are thus extended, more temporal complexity is incurred in consequence. Note that there are $N^L$ possible temporal modulation schemes available for each of the meta-atoms, in which the corresponding information entropy can be expressed as ${\mathrm{ln}}N^L$. On the other hand, the combined operations can be adopted to generate $N \times q$ amplitude-invariant uniform phase states for arbitrary temporal sequences. Hence, the corresponding output information entropy of the meta-atom at the *m*th harmonic frequency can be expressed as ${\mathrm{ln}}Nq$. As a result, the temporal-to-spectral information transition efficiency of the spatiotemporal metasurface induced by the group extensions can be derived as:

7 $$\rho _1(N,L,m) = \ln (Nq)^{PQ}/\ln N^{LPQ} = \ln (Nq)/L\ln N$$

where $P \times Q$ is the number of meta-atoms. To better illustrate the above results, Fig. 3a–d present the theoretically calculated spectral states of a single meta-atom and the corresponding information transition efficiencies with respect to different temporal lengths and input states. We remark that the information transition efficiency is highly affected by the algebraic relation among the harmonic index (*m*), the number of input phase states (*N*) and the length of temporal periodicity (*L*) and decreases as the temporal periodicity (*L*) increases in general.

### Independent controls of multiple harmonics

The group extension schemes demonstrate that the spatiotemporally modulated information (i.e., the input spatiotemporal sequence) can be effectively transformed to extended output spectral responses at a fixed harmonic frequency. Subsequently, we seek the possibility of independent controls of the spectral responses of the spatiotemporal metasurface at multiple harmonics and explore the corresponding information transition efficiencies.

The independent controls of multiple harmonics require the involved spectral responses of each meta-atom to be degenerate and symmetrical at the same time, as sketched in Fig. 2g. To find the required spectral responses of the meta-atom, the combined operator is adopted, which can provide the natural way to generate the required degenerate and symmetrical spectral responses to each meta-atom at multiple harmonics.

Specifically, the temporal sequence ($C$) acted by the combined operations might exhibit sequence degeneracy ($D(C)$) such that $W_N\left( {u,v} \right)C = C$, where $W_N\left( {u,v} \right)$ is not the identity operation. For instance, when $N = L = 8$, there exists a temporal sequence $C = \left( {1,\,1,\,1,\,1,\, - 1, - 1,\, - 1,\, - 1} \right)$ satisfying the relation that $W_8\left( {8,4} \right)\left( {1,\,1,\,1,\,1,\, - 1, - 1,\, - 1,\, - 1} \right) = \left( {1,\,1,\,1,\,1,\, - 1, - 1,\, - 1,\, - 1} \right)$, where $W_8\left( {8,4} \right)$ is not the identity element. Accordingly, $8 \times 4$ distinctive temporal sequences can be generated by the combined operations (rather than $N \times L = 8 \times 8 = 64$), as is sketched in Fig. 2e. In other words, the temporal sequence *C* acted by all elements of the combined operations shares the sequence degeneracy of $D\left( C \right)= (8\, \times\, 8)/(8 \,\times\, 4) = 2$. Consequently, the number of generated temporal sequences must be a divisor of $N \times L$, which can be expressed as $NL/D(C)$. As an important application, it can be verified that the sequence degeneracy must be $D\left( C \right) = 1$ as long as the input phase states (*N*) and temporal periodicity (*L*) are relatively prime. Please refer to the Supplementary Information for more details.

The $NL/D(C)$ distinctive temporal sequences can be used to generate the degenerate and symmetrical spectra responses at multiple harmonics for each meta-atom. In particular, the output spectral response degeneracy of the meta-atom at the *m*th harmonic frequency can be derived as $L/D(C)q$ (see the Supplementary Information for more details). Additionally, the numbers of output symmetrical spectral states of the meta-atom at the involved harmonics are shown to be $N \times q$. For visual demonstration, the spectral responses of sequence *C* when acted by the combined operations are presented in Fig. 2f, in which the degenerate spectral responses at the 1st harmonics are labeled with identical colored symbols. It can be noticed that the spectral responses of the meta-atom are composed of 8 uniform phase states (8 colors), and each phase state shares the common degeneracy of 4.

The symmetry and degeneracy of the spectral responses of the meta-atom can provide guidance for independent controls of multiple harmonics with the spatiotemporal metasurface. For instance, once the spectral symmetry ($Nq$) and degeneracy ($L/D(C)q$) of the meta-atom at each harmonic frequency are obtained, the maximum spectral states for each meta-atom in controlling multiple harmonics is bounded by the product of the two terms as $Nq \times L/D(C)q = NL/D(C)$. Hence, the information transition efficiency of spatiotemporal metasurfaces for independent controls of multiple harmonics would be bounded as:

8 $$\rho _2 \le \max [\ln (NL/D(C))/\ln N^L] \le \ln NL/L\ln N$$

It should be noted that more information can be obtained by analyzing the spectral symmetries and degeneracies of the meta-atoms; extended research on this topic is recommended.

To better illustrate the above concept, Fig. 3e, f presents the upper bound of the spectral states of the meta-atom for independent controls of multiple harmonic responses along with the corresponding information transition efficiency. Note that the upper bound of the spectral states of the meta-atom increases as the temporal periodicity increases; however, the corresponding information transition efficiency decreases in general. It should be noted that the harmonic information transition process of the spatiotemporal metasurface is governed by a trade-off relation between the phase state extensions and independent controls of spectral responses at multiple harmonics. For instance, when $\gcd \left( {L,N} \right) = \gcd \left( {L,m} \right) = 1$, the output spectral responses at the *m*th harmonic contain the maximum phase states of $N \times L$. As a result, the spectral degeneracies at the *m*th harmonic are decreased to the minimum (one), making it impossible to independently control multiple spectral responses that involve the *m*th harmonic frequency.

We remark that it might be possible to adopt the temporal sequences not related by the combined operations to generate extended phase states and independent controls of multiple harmonics. However, the generated spectral responses have different amplitudes in general, and the phase responses cannot readily match the required uniform coverage of 2*π* radians either. Therefore, combined operations are adopted in this work, by which the amplitude-invariant symmetrical and degenerate spectral responses of the meta-atoms are guaranteed.

### Proof-in-principle validation

Among many compelling features, we here consider wavefront engineering of the converted waves to demonstrate the group extension effect and the independent controls of multiple harmonics. A prototype of the spatiotemporal metasurface operated at microwave frequencies is designed and fabricated for proof-in-principle validations, as shown in Fig. 5a, b. The presented metasurface sample consists of 20 × 15 meta-atoms and has an overall size of 140 mm × 105 mm. Each meta-atom is composed of a metal patch etched on a grounded F4B substrate (dielectric constant 2.65 and thickness 3 mm) along with an integrated pin diode. The detailed geometry parameters of the meta-atom are presented in the Supplementary Information. A field programmable gate array (FPGA) is used as the control unit to apply the on-demand spatiotemporal variations to the metasurface, in which the unit modulation duration is *τ* = 0.2 µs. Thus, the modulation frequency can be derived as $f = \frac{1}{{N\tau }} = 1.25\,{\mathrm{MHz}}$. Additionally, simulation results are adopted to verify the effectiveness of the designed metasurface, which can be found in the Supplementary Information.

**Fig. 4:**
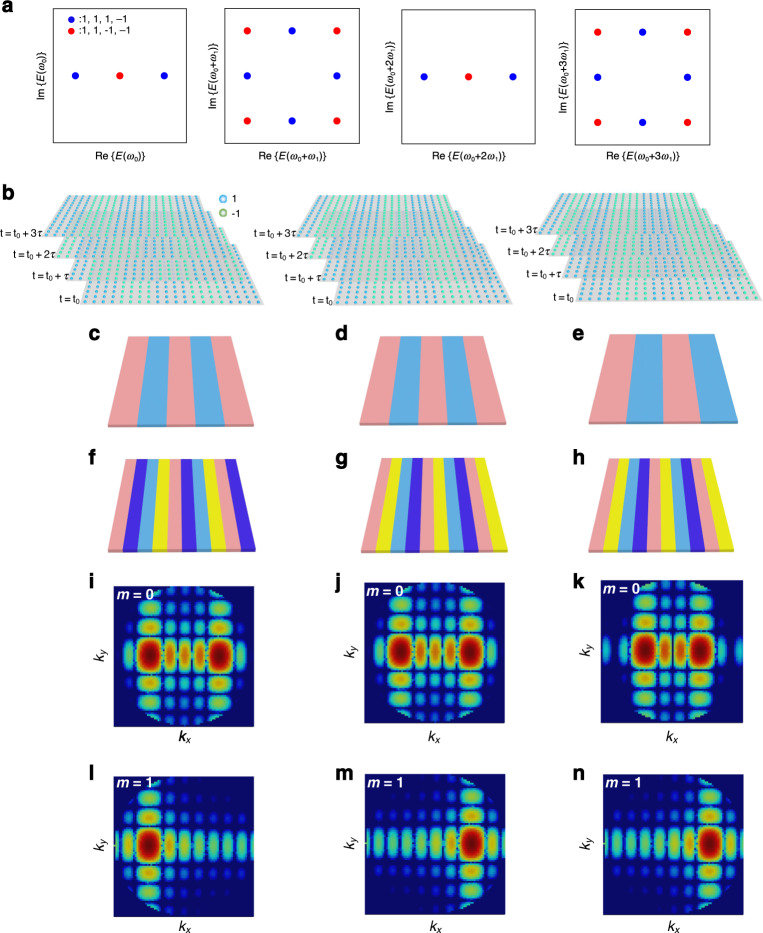
Spatiotemporal modulations of the metasurface and the generated wavevector transitions in the far-field region. **a** The spectral responses of the meta-atom generated by temporal modulations with $N = 2$ and $L = 4$, in which same-colored circles represent the temporal sequences related by combined operations. **b** The three-dimensional representation of the spatiotemporal modulation schemes. **c**–**e** The equivalent phase distributions of the metasurface at the 0th harmonic. **f**–**h** The equivalent phase distributions of the metasurface at the 1st harmonic. **i**–**n** The generated wavevector transitions (radiation patterns) in the far-field regions

When dynamic modulation is adopted for the meta-atom, the possible spectral responses of the meta-atom at each harmonic are presented in Fig. 4a. It is noteworthy that only two sets of sequences (*C*_1_ and *C*_2_) have a non-recurrent temporal periodicity of *L* = 4, in which the corresponding spectral responses at each harmonic are labeled with differently colored points. Here, we choose to adopt the combined operations to the temporal sequence $C_1 = (1,\,1,\, - 1,\,1)$ for demonstration. Evidently, the generated spectral responses are symmetrically distributed on the complex plane at each harmonic (shown in Fig. 4a), in which the corresponding symmetry at each harmonic can be denoted as ${\mathrm{Z}}_2$, ${\mathrm{Z}}_4$, ${\mathrm{Z}}_2$, and ${\mathrm{Z}}_4$, respectively. The obtained output symmetry at each harmonic is consistent with the theoretical prediction of ${\mathrm{Z}}_{N \times q}$, which validates the phase state extension mechanism at the 1st and 3rd harmonic frequencies (with the extension factor of $q = 2$).

Moreover, the above generated spectral states can be used to demonstrate the independent controls of multiple harmonic responses as well. As discussed earlier, the number of independent spectral states at multiple harmonics is bounded by $NL/D(C_1) = 8$. Subsequently, it can be shown that all possible combinations of the 8 spectral responses at the 0th and 1st harmonics are generated, as sketched in Fig. 2g (see the Supplementary Information for more details).

Hence, the harmonic information transition mechanisms of the meta-atom with respect to phase-state extension and independent controls of multiple harmonic responses are demonstrated. More specifically, the information transition efficiency induced by extended phase states at the 1st harmonic can be derived as $\rho _1 = {\mathrm{ln}}Nq/{\mathrm{ln}}N^L = {\mathrm{ln}}4/{\mathrm{ln}}16 = 0.5$, and the information transition efficiency induced by the independent controls of the spectral responses at the 0th and 1st harmonics can be expressed as $\rho _2 = {\mathrm{ln}}NL/{\mathrm{ln}}N^L = {\mathrm{ln}}8/{\mathrm{ln}}16 = 0.75$.

The extended phase states and the independently controlled spectral responses at multiple harmonics induced by the spatiotemporal metasurfaces can be used to engineer the transmission of electromagnetic waves at the required harmonic frequency (frequencies). For validations, three different spatiotemporal modulation schemes are adopted for the metasurface sample.

The three-dimensional representation of the designed spatiotemporal modulation schemes in one temporal period is presented in Fig. 4b, in which the blue and green circles represent the input modulated phase states of “1” and “−1”, respectively. Note that each layer of the parallel slice represents the spatial modulation pattern of the metasurface in one temporal interval, for which four consecutive layers are required to describe the spatiotemporal modulations when the temporal periodicity length is $L = 4$, as shown in Fig. 4b.

Notably, these spatiotemporal patterns are all generated by the permutations and translations of the same sequence of $C_1 = (1,\,1,\, - 1,\,1)$, by which the amplitude-invariant spectral responses are protected. As a result, the corresponding phase distributions at the 0th and 1st harmonics are presented in Fig. 4c–h. According to generalized Snell’s law, the steering angles of the generated radiation patterns with respect to these phase distributions at the 0th harmonic frequency (Fig. 4c–e) can be derived as ${\uptheta}_1 \approx \mp 30^\circ$, ${\uptheta}_2 \approx \mp 30^\circ$, and ${\uptheta}_3 \approx \mp 24^\circ$, respectively. The steering angles of the generated radiation patterns with respect to the phase distributions at the 1st harmonic (Fig. 4f–h) can be derived as ${\uptheta}_4 \approx 30^\circ$, ${\uptheta}_5 \approx 30^\circ$, and ${\uptheta}_6 \approx - 30^\circ$, respectively. In other words, the wavevector transitions of electromagnetic waves induced by the spatiotemporal modulations peak at $k_x \approx \mp \frac{1}{2}k$, $k_x \approx \mp \frac{1}{2}k$, $k_x \approx \mp \frac{2}{5}k$, $k_x \approx \frac{1}{2}k$, $k_x \approx \frac{1}{2}k$, and $k_x \approx - \frac{1}{2}k$. The numerically calculated wavevector transitions (i.e., radiation patterns in the *k*-space) are presented in Fig. 4i–n. We observe that single beam steering centered at $k_x = \mp \frac{1}{2}k$, $k_y = 0$ ($\theta _4 = 30^\circ$, $\theta _5 = 30^\circ$, $\theta _6 = - 30^\circ$) can be effectively realized, as shown in Fig. 4l–n, as generated by the extended four-state gradient phases. Additionally, Fig. 4i–n shows that the far-field energy distributions at the 0th and 1st harmonics can be independently controlled, demonstrating independently controlled harmonic flows.

**Fig. 5:**
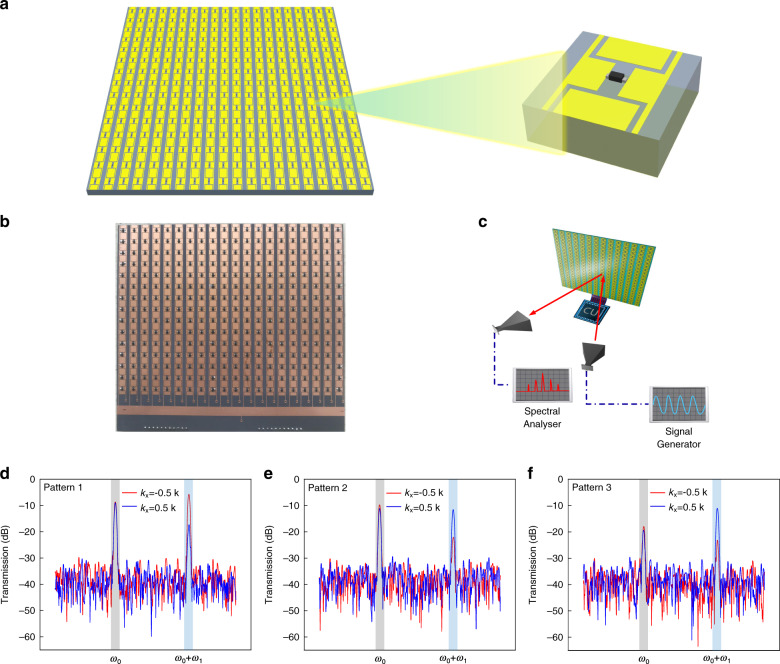
Prototype design and measurement results. **a** Schematic of the designed spatiotemporal metasurface and the meta-atom. **b** Photograph of the fabricated prototype. **c** The measurement setup in an anechoic chamber. **d**–**f** Measured results of the intensity of far-field energy distribution at $k_x = \mp \frac{1}{2}k$, $k_y = 0$ (${\uptheta} = \mp 30^\circ ,\,\varphi = 0^\circ$) at the 0th and 1st harmonics

Limited by the experimental recourses, we measured the intensity of the far-field energy distribution at $k_x = \mp \frac{1}{2}k$, $k_y = 0$ (${\uptheta} = \mp 30^\circ ,\,\varphi = 0^\circ$) only. The measured results with respect to the above three spatiotemporal modulation schemes (Fig. 4b) are illustrated in Fig. 5d–f.

At the first harmonic (${\upomega} = {\upomega}_0 + {\upomega}_1$), it can be noticed that the radiation intensity at $k_x = - \!\frac{1}{2}k$ (red curve) is much higher than the radiation intensity at $k_x = \frac{1}{2}k$ (blue curve), as presented in Fig. 5d. Conversely, the radiation intensity of the first harmonic at $k_x = \frac{1}{2}k$ (blue curve) is much higher the radiation intensity at $k_x = - \!\frac{1}{2}k$ (red curve), as shown in Fig. 5e, f. The above unbalanced wavevector transitions to $k_x = \mp \frac{1}{2}k$ of the radiated waves are consistent with the theoretical predictions and numerical simulations (Fig. 4l–n), which is the manifestation of the extended gradient phases of the spatiotemporal metasurface ($N = 2$ phase states extended as $N \cdot q = 4$ phase states).

Additionally, Fig. 5d, e show that the wavevector transition intensities of the radiated waves at the 1st harmonic (${\upomega} = {\upomega}_0 + {\upomega}_1$) can be switched, in which the wavevector transition intensities at the 0th harmonic (${\upomega} = {\upomega}_0$) are almost invariant. Similarly, the wavevector transition intensities of the radiated waves at the 0th harmonic (${\upomega} = {\upomega}_0$) can be independently switched without affecting the wavevector transition intensities at the 1st harmonic (${\upomega} = {\upomega}_0$), as shown in Fig. 5e, f. The above independent controls of the wavevector transitions of radiated waves at the 0th and 1st harmonics are consistent with the theoretical predictions and numerical simulations (Fig. 4i–n), by which the independent controls of multiple harmonics is demonstrated as well. We observe that the measured spectral responses at the 1st harmonic are centered at *f*_1_ = 1.23 MHz, which deviates ~1.6% from the theoretical perdition (*f*_1_ = 1.25 MHz). The small frequency deviation is likely attributed to the inaccuracy of the switch time of the control unit. Nevertheless, the effects of unbalanced wavevector transitions and independent controls of the 0th and 1st harmonics are clearly observed.

### Upper bound of channel capacity of the spatiotemporal metasurface

The proposed schemes are expected to significantly broaden the application scopes of spatiotemporal metasurfaces and promise important advantages for information-oriented applications such as wireless communications. Accordingly, the maximum information transmission rate (or channel capacity) of the spatiotemporal metasurface, which is featured as the key characteristic for communication systems, should be explored. For the determination of the channel capacity of the spatiotemporal metasurface, we remark that the ideal periodic temporal sequence is time-consuming and is not realizable in general. Thus, the spatiotemporal modulations are usually approximated with finite temporal periods. Accordingly, it is useful to denote the number of repeated temporal periods as *u*, such that the upper bound of the channel capacity of the spatiotemporal metasurface induced by the proposed schemes (group extension and independent controls of multiple harmonics) can be obtained by Shannon’s noiseless channel theorem^30^ as:

9 $$C \le \frac{{I(X)}}{{Lu\tau }} \le PQ\ln NL/Lu\tau {\mathrm{ }}$$

**Fig. 6:**
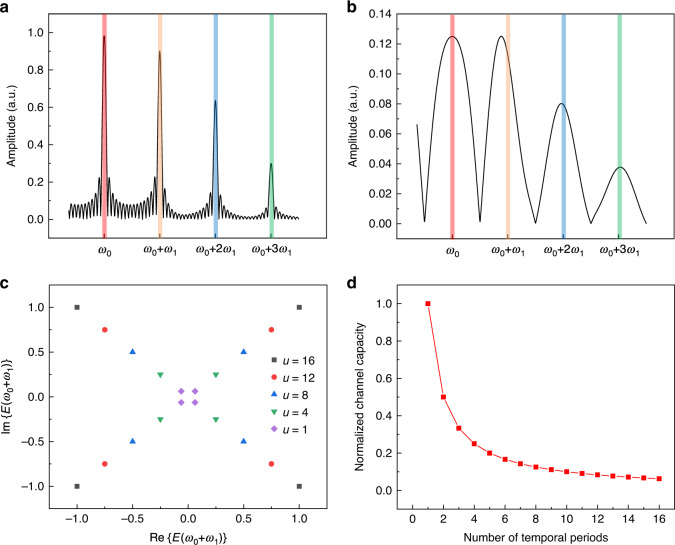
The generated spectral responses of the meta-atom with respect to the finite temporally repeated sequence and the normalized channel capacity of the spatiotemporal metasurface. **a**, **b** The frequency-domain responses of the meta-atom with respect to differently repeated temporal modulations. **c** Constellation diagram of the spectral responses of the meta-atom at the 1st harmonic (${\upomega} = {\upomega}_0 + {\upomega}_1$) generated by the translations of the input sequence $C = (1,\,1,\, - 1,\,1)$ with respect to differently repeated temporal modulations. **d** The upper bound of the normalized channel capacity of the spatiotemporal metasurface induced by the proposed schemes

The term *I*(*X*) is the average amount of self-information provided at the input end of the spatiotemporal metasurface, which is bounded by $PQ{\mathrm{ln}}NL$. The above inequality indicates that the upper bound of the channel capacity varies inversely as the number of temporal periods increases, as presented in Fig. 6d. Notably, the number of temporal periods should not be too small to avoid a deviation in the harmonic frequency, as shown in Fig. 6b. Additionally, it can be observed from Fig. 6a, b that the converted fields are more diffused in the frequency-domain when the temporal period decreases, causing the intensity of the spectral responses of the meta-atom to be decreased at the discrete harmonics. In addition, a decrease in the temporal periods causes the overall spectral energy to be decreased as well, such that the magnitude of the converted fields of the meta-atom at the harmonics scales with the number of temporal periods (see the Supplementary Materials for more information). For instance, Fig. 6c shows the calculated constellation diagram of the spectral responses generated by the translations of the input sequence $C = (1,\,1,\, - 1,\,1)$ with respect to differently repeated temporal periods. We clearly observe that the spectral response amplitudes of the converted fields increase linearly as u increases. Therefore, it is demonstrated that the channel capacity and the intensity of converted fields of the spatiotemporal metasurface are governed by a trade-off relation.

## Discussion

We have shown that the harmonic information transitions of spatiotemporal metasurfaces are closely related by the temporal periodicity (*L*), modulation states (*N*), harmonic index (*m*), and temporal repetition factor (*u*). In principle, once the number of input phase states and the temporal periodicity of the metasurface are specified, the manipulation of electromagnetic waves can be effectively realized by engineering the spatiotemporal sequences. Notably, there might exist unwanted scenarios in which the intensity of converted spectral responses of the meta-atoms degenerates to zero, making the radiated waves vanish. However, when certain physical conditions are imposed, the non-vanished harmonic flows are effectively protected, unaffected by the modulation schemes. Specifically, for the simplest two-phase-state spatiotemporal metasurface, it can be shown that all spectral flows (from the 0th to *L* − 1th harmonic frequencies) must exist when the temporal periodicity (*L*) of the spatiotemporal modulations is an odd prime. Detailed proof can be found in the Supplementary Information. More intriguingly, it can be shown that the output spectral responses of the spatiotemporal metasurface can help prove and visualize Fermat’s little theorem, which might give us more clues to understand the harmonic information transitions of the spatiotemporal metasurfaces in return. For more discussion, please refer to the Supplementary Information.

We remark that optimization algorithms such as particle swarm optimization (PSO) and the genetic algorithm (GA) can be adopted to design spatiotemporal metasurfaces as well. However, the present theory establishes the natural way to generate the required spectral responses of the meta-atom at arbitrary harmonic frequency (frequencies), by which the time-consuming and heavy-computational requirements of the optimization algorithms can be greatly alleviated. Moreover, the obtained results, such as the non-vanished spectral responses of the meta-atom, can be directly used to guide the design of spatiotemporal metasurfaces, by which the unnecessary optimization steps can be greatly reduced.

In summary, the proposed theory establishes a quantitative framework to characterize the information transition capabilities of spatiotemporal metasurfaces, providing deeper physical insights into spatiotemporal metasurfaces from an information perspective and offering new approaches to facilitate analysis and design. The presented framework and obtained results, with wide-ranging spectral applicability, are helpful to lay the groundwork for future research into the regime of information-based spatiotemporal metasurfaces and are expected to enable new information-oriented applications, including cognitive harmonic wavefront engineering, intelligent computational imaging, and 6th generation (6G) wireless communications.

## Materials and methods

### Experimental measurement

A proof-of-concept experiment is carried out in a microwave anechoic chamber, for which the measurement setup is illustrated in Fig. 5c. A linearly polarized horn antenna working in the X band provides monochromatic plane-wave excitation at $f = 10.6\,{\mathrm{GHz}}$. Another horn antenna is used to receive broadband scattered waves via a spectrum analyzer. An FPGA control unit (Cyclone IV) is exploited to provide the biasing voltages for the spatiotemporal metasurface, in which each column is dynamically engineered with the preloaded spatiotemporal sequences as presented in Fig. 4b.

## Supplementary information

Supplementary Information for Harmonic information transitions of spatiotemporal metasurfaces (Revision)
