# Supplementary material for: Harmonic information transitions of spatiotemporal metasurfaces
Source: Light Sci Appl. 2020 Dec 14;9:198. doi: 10.1038/s41377-020-00441-1 (PMC7736919; doi:10.1038/s41377-020-00441-1)
Supplement: Supplementary file 1 — Supplementary Information for Harmonic information transitions of spatiotemporal metasurfaces (Revision) [file 41377_2020_441_MOESM1_ESM.docx]

**Supplementary Information for**

**Harmonic information transitions of spatiotemporal metasurfaces**

Haotian Wu^1,2^, Xin Xin Gao^1,2^, Lei Zhang^1,2^, Guo Dong Bai^1,2^ , Qiang Cheng^1,2^, Lianlin Li^3^, and Tie Jun Cui^1,2*^

^1^ State Key Laboratory of Millimeter Waves, Southeast University, Nanjing 210096, China

^2^ Institute of Electromagnetic Space, Southeast University, Nanjing 210096, China

^3^ State Key Laboratory of Advanced Optical Communication Systems and Networks, Department of Electronics, Peking University, Beijing 100871, China

^*^Email: [tjcui@seu.edu.cn](mailto:tjcui@seu.edu.cn)

This Supplementary Information includes the following materials and figures:

1. The characteristics of the generated sequences when *N* and *L* are relatively prime.
2. The details of geometry and responses of meta-atom.
3. The simulation results of the radiation patterns generated by designed metasurface.
4. The group extension factor of the spatiotemporal metasurface.
5. The spectral response degeneracy of the meta-atom generated by the combined operator.
6. Characteristic of spectral responses of the meta-atom generated by the temporal sequence of *C*_1_.
7. Characteristics of spectral response of the meta-atom with respect to the number of temporal periods.
8. The proof of non-vanishing property of the converted field and the associated Fermat’s little theorem.
9. The proof of irreducibility of special polynomials by Eisenstein’s criterion.

**1. Characteristics of the generated sequences when *N* and *L* are relatively prime**

The permutation operations can introduce a natural equivalent relation, in which the temporal sequence related by the permutations can be grouped together as an equivalent class, and each class contains *N* distinctive elements. Accordingly, the total number of the sequences must be multiples of *N*. Similarly, it can be verified that the number of the sequences must be multiples of *L* as well when the translation operations are considered. Consequently, the numbers of the generated sequences must be multiples of *N* and *L* simultaneously. Therefore, the number of the generated sequence must be $N\times L$ if *N* and *L* are relatively prime. That is to say, the corresponding degeneracy of the temporal sequence must be $D\left( C \right)=1$.

**2. Geometry and electromagnetic responses of the meta-atom**

Figure S1a shows the geometry and relevant parameters of the meta-atom. Four patches are etched on the F4B dielectric substrate, with the designed geometrical parameters as: *a* = 7 mm, *b*_1_ = 3.7 mm, *b*_2_ = 4.9 mm, *c* = 3 mm, *d*_1_ = 1.1 mm, and *d*_2_ = 5 mm. A PIN-diode (SMP-1320) is employed to connect the adjacent patches. The equivalent circuit model^1^ of the PIN diode is illustrated in Figure S1b, in which *R* = 0.5 Ω, *C* = 0 pF, and *L* = 0.75 nH in the ‘ON’ state; while *R* = 0 Ω, *C* = 0.24 pF, and *L* = 0.5 nH in the ‘OFF’ state.


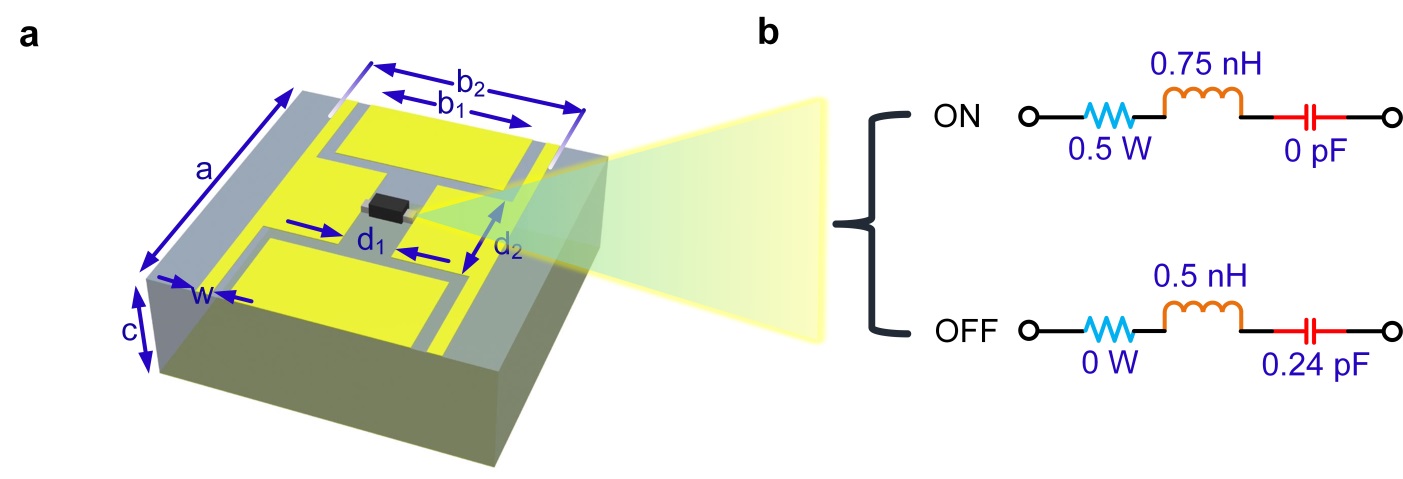


**Figure S1. a,** The top view of the meta-atom with detailed geometrical parameters. **b,** The equivalent circuit models of the PIN diode biased in the “ON” and “OFF” states.

The amplitudes and phases of the reflection coefficients pertaining to the “ON” and “OFF” states of the PIN diode are illustrated in Figures S2a and b, respectively. We observe that the phase difference is approximately 180^o^ at the frequency band from 10.1 to 10.7 GHz, and the corresponding amplitude is above 0.85. Accordingly, the complex amplitude of the meta-atom can be switched between these two states with the bias voltage manipulated by the control unit.


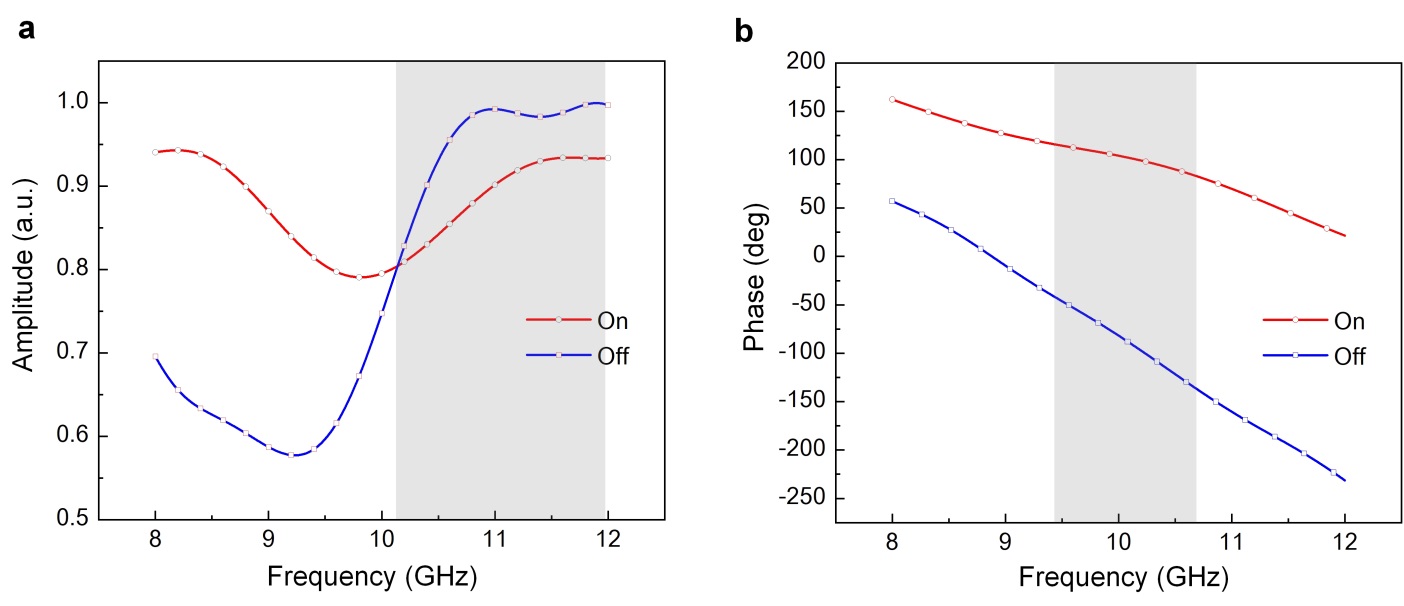


**Figure S2.** Numerically simulated reflection responses of the meta-atom in the “ON” and “OFF” states. **a,** The amplitude. **b,** The phase.

**3. The simulation results of the radiation patterns generated by designed metasurface.**

Three different modulation schemes are adopted to demonstrate the wavefront engineering of the designed metasurface. In these modulation schemes, the theoretical calculated radiation patterns (Figures S3a-c) and the numerical simulated radiation patterns (Figures S3d-f) are both presented. The commercial software of CST Microwave Studio is adopted for the modeling of the metasurface and simulation of the generated radiation patterns. In the first modulation scheme, the pin diodes of the metasurface are all set be at the ‘off’ states, by which the normally incident beam will be reflected back by the metasurface. In the second modulation scheme, the states of the pin diodes are set as periodic pattern as ‘on, on, on, on, off, off, off, off, on…’. Consequently, the normally incident beam will be deflected to the two symmetrical angles, and the deflection angles can be derived by generalized Snell’s law as $\theta=\mp\sin^{-1}\left( \frac{\lambda}{\Gamma} \right)\approx\mp30^{\circ}$. The term $\Gamma$ represents the spatial periodicity of the phase distribution of the metasurface. In the third modulation scheme, the states of the pin diodes are set as periodic pattern as ‘on, on, on, on, on, off, off, off, off, off, on…’. Similarly, the normally incident beam will be deflected to the two symmetrical angles, and the deflection angles can be derived as $\theta=\mp\sin^{-1}\left( \frac{\lambda}{\Gamma} \right)\approx\mp24^{\circ}$. The calculated and simulated radiation patterns with respect to these modulation schemes are presented in Figures S3a-c and Figures S3d-f respectively. It can be noticed that the simulated results are in consistent with the theoretical predictions, which validates the design of the metasurface.


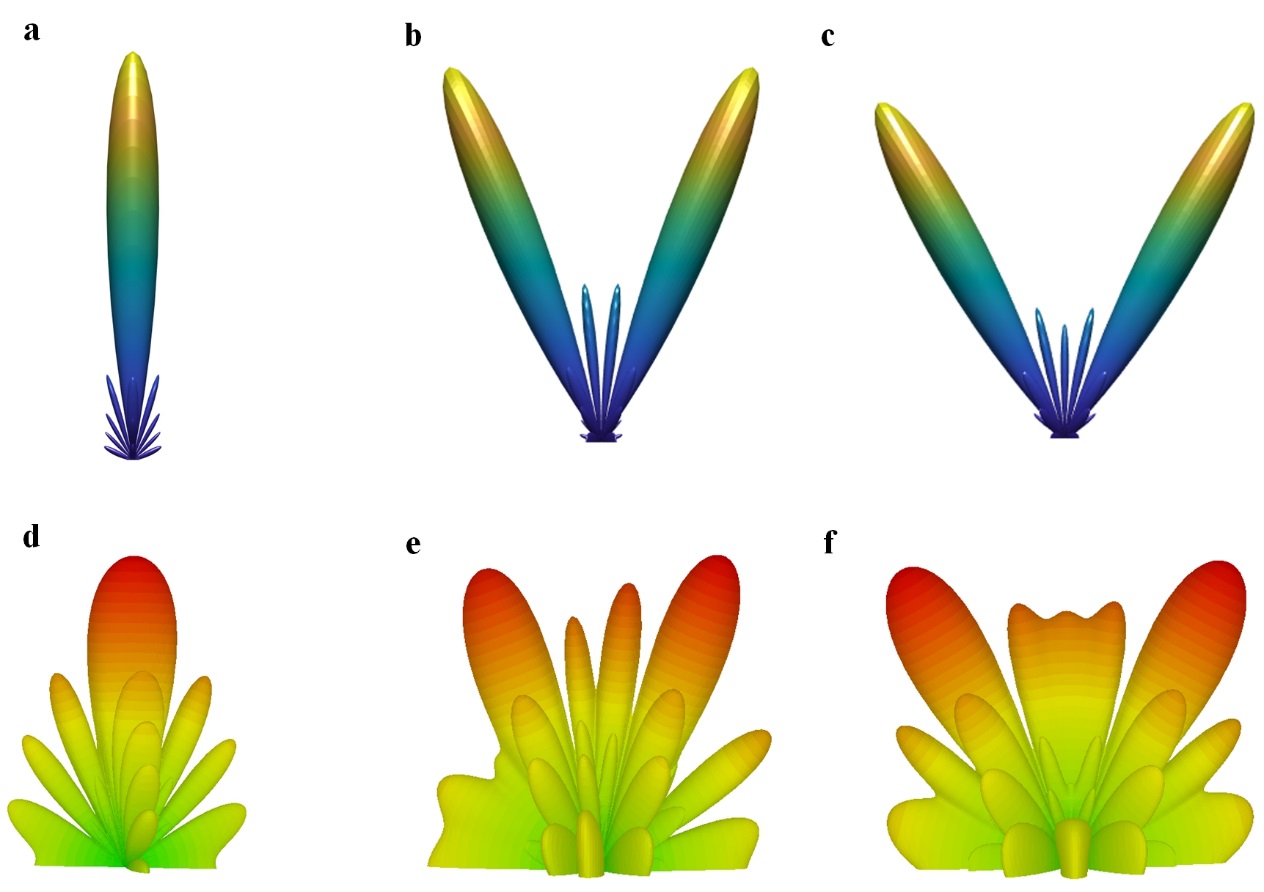


**Figure S3.** The calculated results (**a**-**c**) and simulated results (**d**-**f**) of the radiation patterns generated by the designed metasurface with respect to different modulation schemes.

**4. The group extension factor of the spatiotemporal metasurface**

It has been shown that frequency-dependent output phase state of ${\zeta_{N}^{a}\times\zeta}_{L}^{-bm}$ will be generated when adopted by the combined operator $W_{N}(a,b)$. As a result, the total number of spectral responses of each meta-atom at the *m*^th^ harmonic is determined by the number of distinctive phase shifted states generated by ${\zeta_{N}^{a}\times\zeta}_{L}^{-bm}$, in which a and b can be arbitrary integers ($a,b\mathbb{\in Z}$). One way to solve this problem requires knowledge of group theory and algebraic number theory, in which the detailed analysis is listed below.

Firstly, it is helpful to simplify the problem and analyze the phase shifted states when only one of the term ($\zeta_{N}^{a}$or $\zeta_{L}^{-bm}$) is considered. For the term $\zeta_{N}^{a}$, it is evident that *N* phase states ($\zeta_{N}^{0}, \zeta_{N}^{1}\ldots\zeta_{N}^{N-1}$) uniformly covering $2\pi$ radians will be generated when the parameter ‘a’ runs from 0 to *N*-1. As for the term $\zeta_{L}^{-bm}$, it could be reformulated as:$\zeta_{L}^{-bm}=\exp\left( -j2\pi\frac{m}{L}b \right)=\exp\left( -j2\pi\frac{m/gcd(L,m)}{L/gcd(L,m)}b \right)$. Noted that common divisor term $gcd(L,m)$ is introduced to make sure that the fraction $\frac{m/gcd(L,m)}{L/gcd(L,m)}$ is the simple fraction (i.e. the numerator and denominator are relatively prime).

Subsequently, a relevant proposition in algebraic number theory should be referred, which says that ***a pair (x, y) of integers is relatively prime if and only if there are integers r and s such that*** $\boldsymbol{rx+sy=1}$***.*** That is to say, there must exist a pair of integers r and s such that $r\times m/gcd(L,m)+s\times L/gcd(L,m)=1$, since $m/gcd(L,m)$ and $L/gcd(L,m)$ are relatively prime. This relation can be reformulated as $r\times\frac{m/gcd(L,m)}{L/gcd(L,m)}+s=\frac{1}{L/gcd(L,m)}$, where r can be solved as $r=(\frac{1}{L/gcd(L,m)}-s)\times\frac{L/gcd(L,m)}{m/gcd(L,m)}$. Next, let $b=-c\times r$ (c is an arbitrary integer) and substitute b and r in the term $\zeta_{L}^{-bm}$, by which the term $\zeta_{L}^{-bm}$ can be reformulated as: $\zeta_{L}^{-bm}=exp \left( j2\pi\frac{m/gcd(L,m)}{L/gcd(L,m)}cr \right)=exp[j2\pi c(\frac{1}{\frac{L}{\gcd\left( L,m \right)}}-s)]=exp[j2\pi(\frac{c}{L/gcd(L,m)})]$.

Above analysis indicates that taking –r as a unit step for $\zeta_{L}^{-bm}$ will generate phase shift of $exp[j2\pi(\frac{1}{L/gcd(L,m)})]$. As a result, $L/gcd(L,m)$ uniform phase states ($\zeta_{L/gcd(L,m)}^{0}, \zeta_{L/gcd(L,m)}^{1}\ldots\zeta_{L/gcd(L,m)}^{L/gcd(L,m)-1}$) would be generated when *c* runs from 0 to $L/gcd(L,m)-1$. In other words, it is demonstrated that the phase shifted states generated by $\zeta_{L}^{-bm}$ are equivalent to the phase shift states generated by $\zeta_{L/gcd(L,m)}^{c}$. Therefore, we could analyze the phase shift states generated by ${\zeta_{N}^{a}\times\zeta}_{L/gcd(L,m)}^{c}$ instead, for which the obtained results are equivalent to case of ${\zeta_{N}^{a}\times\zeta}_{L}^{-bm}$.

Next, one relevant proposition in group theory should be referred as well, in which the proposition says that: ***let u and v be integers that are not both zero, and let w be their greatest common divisor (gcd), the positive integer that generates the subgroup*** $\boldsymbol{S}\mathbb{=Z}\boldsymbol{u}\mathbb{+Z}\boldsymbol{v}$ ***of the additive group of integers (***$\mathbb{Z,+}$***), so*** $\mathbb{Z}\mathbf{w=}\mathbb{Z}\mathbf{u+}\mathbb{Z}\mathbf{v}$**.** In other words, the range of $a\times u+c\times v$ ($a,c=\ldots-2,-1,0,1,2\ldots$) are integers of multiples of $gcd(u,v)$.

According to above proposition, we could choose to let $u=\frac{L}{gcd(L,m)}$ and $v=N$, such that: $\frac{L}{gcd(L,m)}a+Nc= gcd(\frac{L}{\gcd\left( L,m \right)},N)\times p$, where *p* is an arbitrary integer. This relation can be reformulated by dividing $\frac{L}{gcd(L,m)}\times N$ on both sides as: $\frac{a}{N}+\frac{c}{L/gcd(L,m)}=\frac{\frac{L}{gcd(L,m)}a+Nc}{NL/gcd(L,m)}=\frac{gcd(\frac{L}{\gcd\left( L,m \right)},N)}{NL/gcd(L,m)}p=\frac{1}{\mathrm{lcm}(N,L/gcd(L,m))}p$. As a result, the term ${\zeta_{N}^{a}\times\zeta}_{L/gcd(L,m)}^{c}$ can be expressed as: ${\zeta_{N}^{a}\times\zeta}_{L/gcd(L,m)}^{c}=exp(j2\pi(\frac{a}{N}+\frac{c}{L/gcd(L,m)}))=exp[j2\pi(\frac{1}{\mathrm{lcm}(N,L/gcd(L,m))}p)]$.

Evidently, above representation of ${\zeta_{N}^{a}\times\zeta}_{L/gcd(L,m)}^{c}$ implies that $\mathrm{lcm}(N,L/gcd(L,m))$ phase shifted states uniformly covering $2\pi$ radian will be generated when *p* runs from 0 to $\mathrm{lcm}\left( N,\frac{L}{\gcd\left( L,m \right)} \right)-1$. Noted that the total number of generated phase states ($\mathrm{lcm}(N,L/gcd(L,m))$) must be multiples of *N*, which could be expressed as $N\times q$. Hence, the term q can be derived as:

$q=lcm(N,L/\gcd(L,m))/N=L/[\gcd(L,m)\cdot\gcd(N,L/\gcd(L,m))]$ (1)

The term *q* can be further denoted as group extension factor, for which it determines the structure of the extended group $Z_{N}\times Z_{q}$ that generates the $N\times q$ uniform phase states, as shown in Figures 2b and c.

**5. The spectral response degeneracy of meta-atom generated by the combined operator**

It has been revealed that $NL/D(C)$ distinctive temporal modulation schemes of the meta- atom will be generated when acted by the combined operations. Additionally, it has been shown that $N\times q$ distinctive phase states of the meta-atom will be generated when acted by the combined operations. Due to the symmetry of the combined operations, the generated spectral responses must share the common degeneracy. Therefore, the spectral response degeneracy of the meta-atom at the $m^{\mathrm{th}}$ harmonic degeneracy generated by the combined operations is determined by the quotient of these two terms ($NL/D\left( C \right)$ and $N\times q$ as: $[NL/D\left( C \right)]/(N\times q)=L/D(C)q$.

**6. Characteristic of spectral responses of the meta-atom generated by the temporal sequence of *C*_1_**

It is noteworthy that the actions of the combined operations $W_{2}(a,b)$ can be divided into two sets: the subgroup $W_{2}(0,b_{1})$ that corresponds to the translation operations only, and the coset $W_{2}(1,b_{2})$ that involves both permutations and translations. Accordingly, the temporal sequence acted by the translation operation $W_{2}(0,b_{1})$ will generate the phase shifts of 0 and $b_{1}\pi/4$ at the 0^th^ and 1^st^ harmonics, respectively; while the temporal sequence acted by the coset $W_{2}(1,b_{2})$ will generate the phase shifts of $\pi$ and $b_{2}\pi/4+\pi$ at the 0^th^ and 1^st^ harmonics, respectively. Hence, it can be verified that all possible combinations of the spectral responses at the two harmonics can be generated, which can be visualized from Figure 2g.

**7. Characteristics of spectral response of the meta-atom with respect to the number of temporal periods.**

The complex amplitude of the spectral response of the meta-atom at the *m*^th^ harmonic frequency is in proportion to the *m*^th^ Fourier component of the input temporally modulated responses. Specifically, the *m*^th^ Fourier component (denoted as $\alpha_{m}$ here) of the temporal responses of the meta-atom can be expressed as $\alpha_{m}=\int_{0}^{uT} C_{u}(t)e^{-jmwt}dt$, where $C_{u}(t)$ is the temporal responses of the meta-atom in u periods. Due to the periodicity of the temporal responses and the oscillating factor $e^{-jmwt}$, the expression of $\alpha_{m}$ can be re-expressed as $\alpha_{m}=u\int_{0}^{T} C(t)e^{-jmwt}dt$, where $C(t)$ is the temporal responses of the meta-atom in one period. Therefore, it is proofed that spectral response of the meta-atom at the *m*^th^ harmonic frequency is in proportion to the number of repeated temporal periods (u) as long as *u* being set as a positive integer.

**8. The proof of non-vanished harmonic flow and Fermat’s little theorem**

The temporal sequence of the meta-atom in a period with input states of *N*=2 can be expressed by the time-shifted combination of 1’s and -1’s with the form $C=(C^{0},C^{1}\ldots C^{L-1})$. Next, we suppose that the output spectral response at the *m*^th^ harmonic is zero, which in turn can be expressed with a tensor form as $\Omega_{i}\left( m \right)C^{i}=0$. Due to the amplitude-invariant property of the translation operator, the spectral response generated by any translation of the sequence *C* will be fixed to zero as well, which can be expressed as:$\Omega_{i}\left( m \right)T^{i}\left( a \right)C=0$. The term $T^{i}\left( a \right)C$ represents the *i*^th^ element of sequence *C* acted by the $T(a)$ operation. We remark that the translation operations can generate *L* distinctive temporal sequence. That is to say, the term $\Omega_{i}\left( m \right)T^{i}\left( a \right)\left( C \right)=0$ contains *L* linear equations, which can be rewritten with the tensor product form as: $D_{j}^{i} \Omega^{j}(m)=0$, where $D_{j}^{i}=T^{i}\left( j \right)C$. The term $D_{j}^{i}$ represents the matrix element of the *L*-dimensional matrix composed of 1’s and -1’s.

To simplify the analysis, another *L*-dimensional matrix (*E*) is introduced, in which the matrix elements are all equal to 1. It can be verified that the tensor product relation of $E_{j}^{i} \Omega^{j}(m)=0$ is hold as well. Due to the linearity of matrix product, above two matrix equations can be added, and denoted as: $G_{j}^{i} \Omega^{j}\left( m \right)=0$, where $G_{j}^{i}$ is defined as ${G_{j}^{i}=0.5*(D}_{j}^{i}+E_{j}^{i})$. It is noteworthy that the matrix *G* is a circulant Toeplitz matrix^2^ made of 0’s and 1’s. The rank of such matrix can be derived as *L*-*d*, where *d* is the degree of the greatest common divisors of $f(x)$ and $x^{L}-1$, and $f(x)$ is the associated polynomial of a Toeplitz matrix that:

$f(x)=G_{0}^{0}+G_{1}^{0}x+...+G_{L\text{-}1}^{0}x^{L-1}$ (2)

Noted that the term $x^{L}-1$ can be factored out as: $x^{L}-1=(x-1)(x^{L-1}+x^{L-2}+\ldots1)$. It can be verified by Eisenstein’s criterion that $x^{L-1}+ x^{L-2}+\ldots+1$ is irreducible over rational numbers when *L* is a prime (see Supplementary Note 9 for more details). Additionally, due to the temporal non-recurrence of input sequence, the substitution of *x*=1 to the polynomial *f*(x) will not generate zero either. Consequently, there is no nontrivial common divisor between *f*(*x*) and $x^{L}-1$, such that the rank of the matrix *G* must be *L*-0=*L*. In other words, *G* is proofed to be a real valued non-degenerate matrix. Therefore, the generated spectral responses of the meta-atom cannot be zero, for which $\Omega\left( m \right)$ is a non-zero vector.

To verify the above derivations, we calculate the spectral responses of the meta-atom at the harmonic frequencies. It can be verified from equation (5) in the main manuscript that the spectral response distributions at the non-zero harmonics (1^st^ harmonic, 2^nd^ harmonic…, *L*-1^th^ harmonic) are the same only up to a constant multiples as long as *L* is prime, such that only the 0^th^ and 1^st^ spectral response distributions are required to be analyzed. Accordingly, the spectral response distributions of the meta-atom generated by all of the temporal sequences with the same temporal periodicity are plotted in Figures S4a-b, from which we can clearly observe that no zero spectral response is generated.

Now, it has been established that the intensity of the converted spectral responses of the meta-atoms are closely related to the abstract number relation between the input states and temporal periodicity. Furthermore, we will dig deeper and show that the output spectral responses of the spatiotemporal metasurface can help proof and visualize the Fermat’s little theorem geometrically, which in turn might give us clues to generate non-vanished radiation patterns for more general cases.

Firstly, noted that the combined operations introduce a natural equivalence relation that the sequences related by the combined operations can be grouped as an equivalent class. Accordingly, all of the possible input combinations can be partitioned, for which an equivalence relation can be used to partition a set. Subsequently, suppose that the temporal periodicity (*L*) of the input sequence of the meta-atom is a prime number, and the number of input states is not multiples of *L*. In this scenario, the total number of non-recurrent sequences can be derived as $N^{L}-N$. Additionally, it has been established that each equivalent class contains $N\times L$ distinctive input sequences, such that the total number of non-recurrent sequences must be multiple of $N\times L$. In other words, we obtain the algebraic relation that:

$N^{L}-N\equiv0\mathrm{mod}NL$ (3)

where *L* is a prime number, and $N\mathrm{mod}L\neq0$. Surprisingly, the above equation is the variation of the Fermat’s little theorem. Geometrically, the Fermat’s little theorem can be visualized that the $N^{L}-N$ spectral responses of the meta-atom at the nontrivial harmonics (1^st^ harmonic, 2^nd^ harmonic…,*L*-1^th^ harmonic) share the rotational symmetry of $Z_{NL}$, as shown in Figure S4c. And the spectral responses of zero’s have the degeneracy of $D\equiv0 mod L$. More intriguingly, the obtained modulo zero degeneracy might imply the possibility that there is no allowed zero spectral response for the meta-atom when the above conditions are met, by which the extended researches on this topic are recommended.


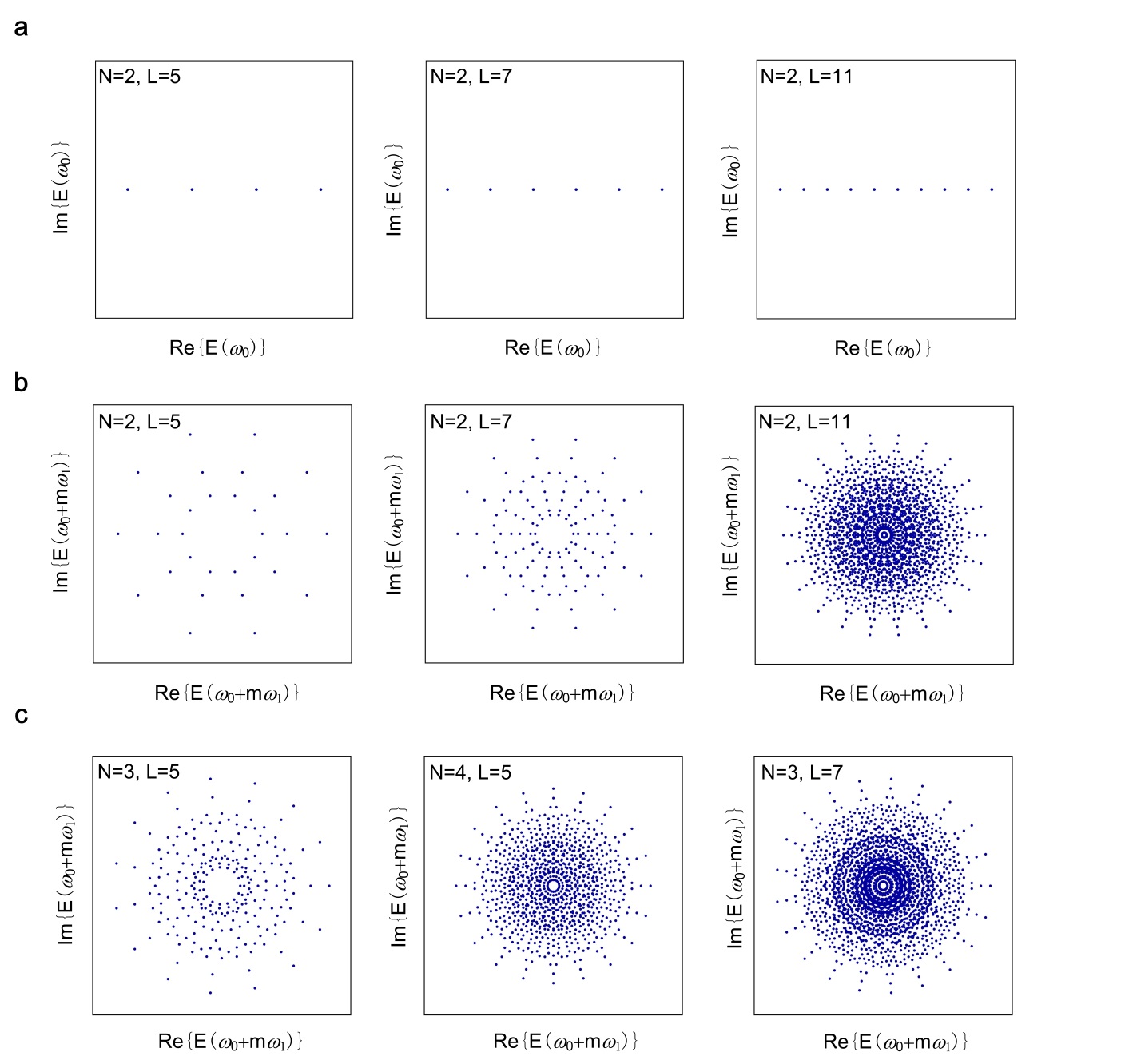


**Figure S4.** Harmonic amplitude patterns for the converted field with respect to temporal periodicity and number of states. **a-b,** Trivial and nontrivial harmonic amplitude distributions with *N* equals 2 and *L* being an odd prime. **c,** Nontrivial harmonic amplitude distributions when *L* is a prime, and *N* is not modulo *L.*

**9. Proof of irreducibility of polynomial** $\boldsymbol{x}^{\boldsymbol{L-1}}\boldsymbol{+} \boldsymbol{x}^{\boldsymbol{L-2}}\boldsymbol{+\ldots+1}$ **by Eisenstein's criterion**

Firstly, the polynomial $x^{L-1}+ x^{L-2}+\ldots+1$ can be rewritten as:

$x^{L-1}+x^{L-2}+...+1=\frac{x^{L}-1}{x-1}$ (4)

Subsequently, the substitution of *x* + 1 for *x* can be adopted, such that the above equation can be reformulated as:

$\frac{(x+1)^{L}-1}{x}=x^{L-1}+\left(\begin{matrix} L \\ L-1 \end{matrix}\right)x^{L-2}+...+\left(\begin{matrix} L \\ 2 \end{matrix}\right)x+\left(\begin{matrix} L \\ 1 \end{matrix}\right)$ (5)

Evidently, all of those non-leading coefficients are divisible by *L* by the properties of binomial coefficients. In addition, the constant coefficient is equal to *L*, and thus is not divisible by *L*^2^_._ Consequently, the Eisenstein’s criterion is fulfilled, indicating that the polynomial $x^{L-1}+ x^{L-2}+\ldots+1$ is irreducible over the rational numbers as long as *L* is a prime number. In other words, the polynomial $x^{L-1}+ x^{L-2}+\ldots+1$ cannot be factorized into the products of non-constant.

**References**

1. Ma, Q. et al. Smart metasurface with self-adaptively reprogrammable functions. *Light: Science & Applications* **8**, 98 (2019).

2. Ingleton, A. W. The Rank of Circulant Matrices. *Journal of the London Mathematical Society* **s1-31**, 445-460 (1956).
